# The Nucleoprotein and Phosphoprotein of Peste des Petits Ruminants Virus Inhibit Interferons Signaling by Blocking the JAK-STAT Pathway

**DOI:** 10.3390/v11070629

**Published:** 2019-07-08

**Authors:** Pengfei Li, Zixiang Zhu, Xiangle Zhang, Wen Dang, Linlin Li, Xiaoli Du, Miaotao Zhang, Chunyan Wu, Qinghong Xue, Xiangtao Liu, Haixue Zheng, Yuchen Nan

**Affiliations:** 1 Department of Preventive Veterinary Medicine, College of Veterinary Medicine, Northwest A&F University, Yangling 712100, China; 2 State Key Laboratory of Veterinary Etiological Biology, National Foot and Mouth Diseases Reference Laboratory, Key Laboratory of Animal Virology of Ministry of Agriculture, Lanzhou Veterinary Research Institute, Chinese Academy of Agricultural Sciences, Lanzhou 730046, China; 3 China Institute of Veterinary Drug Control, Beijing100081, China

**Keywords:** peste des petits ruminants virus, nucleoprotein, phosphoprotein, interferon, JAK/STAT

## Abstract

Peste des petits ruminants virus (PPRV) is associated with global peste des petits ruminants resulting in severe economic loss. Peste des petits ruminants virus dampens host interferon-based signaling pathways through multiple mechanisms. Previous studies deciphered the role of V and C in abrogating IFN-β production. Moreover, V protein directly interacted with signal transducers and activators of transcription 1 (STAT1) and STAT2 resulting in the impairment of host IFN responses. In our present study, PPRV infection inhibited both IFN-β- and IFN-γ-induced activation of IFN-stimulated response element (ISRE) and IFN-γ-activated site (GAS) element, respectively. Both N and P proteins, functioning as novel IFN response antagonists, markedly suppressed IFN-β-induced ISRE and IFN-γ-induced GAS promoter activation to impair downstream upregulation of various interferon-stimulated genes (ISGs) and prevent STAT1 nuclear translocation. Specifically, P protein interacted with STAT1 and subsequently inhibited STAT1 phosphorylation, whereas N protein neither interacted with STAT1 nor inhibited STAT1 phosphorylation as well as dimerization, suggesting that the N and P protein antagonistic effects were different. Though they differed in their relationship to STAT1, both proteins blocked JAK-STAT signaling, severely negating the host antiviral immune response. Our study revealed a new mechanism employed by PPRV to evade host innate immune response, providing a platform to study the interaction of paramyxoviruses and host response.

## 1. Introduction

Peste des petits ruminants (PPR), a severe contagious viral disease of domestic and wild small ruminants, particularly affects goats and sheep [1]. The disease was first reported in the Ivory Coast area of West Africa in 1942 [2]. Since then, PPR has markedly spread from the Ivory Coast to numerous other regions, including countries in Africa, the Middle East, Asia, and Europe [3,4,5,6]. Currently, PPR represents a global threat to about 62.5% of goat and sheep populations and is considered by the goat and sheep industry to be an economically important infectious disease worldwide [7].

The causative agent of PPR, peste des petits ruminants virus (PPRV), belongs to the genus *Morbillivirus* within the family *Paramyxoviridae* [8]. Peste des petits ruminants virus is a non-segmented, negative-sense, single-stranded RNA virus with a genome length of about 15 kb. The viral genome contains six genes encoding eight proteins that include nucleoprotein (N), phosphoprotein (P), matrix protein (M), fusion protein (F), hemagglutinin membrane glycoprotein (H), and large protein (L) structural proteins, as well as non-structural W and C/V proteins [7]. The V and W proteins are the co-transcripts of the P gene due to the one and double G insertion at the editing site, respectively, during the viral transcription. Meanwhile, mRNA coding for the C protein is also generated from the P protein-encoding gene, but through transcription that generates mRNA that encodes an alternate open reading frame [7].

Type I interferons (IFNs) (including IFN-α and IFN-β) and Type II IFN (IFN-γ) are induced during most viral infections and are responsible for restriction of viral replication in vivo [9]. Type I IFNs directly activate the antiviral response in infected or uninfected cells through upregulation of a series of IFN-stimulated genes (ISGs) that ultimately inhibit viral replication [10,11]. Type I IFNs (IFN-α and IFN-β) bind to IFN-α/β receptors, which are composed of interferon-α/β receptor 1 (IFNAR1) and IFNAR2 subunits, to induce autophosphorylation of janus kinase 1 (JAK1) and tyrosine kinase 2 (TYK2). Actions of these activated kinases further lead to phosphorylation of signal transducers and activators of transcription (STAT) that include STAT1 and STAT2 [12,13]. Subsequently, phosphorylated STAT1 and STAT2 form heterodimers that bind to IFN-regulatory factor 9 (IRF9) to form IFN-stimulated gene factor 3 (ISGF3) [14,15], which functions as a transcriptional factor and is imported into the nucleus to bind to IFN-stimulated response elements (ISREs), initiating expression of various ISGs [10,11].

Type II IFN (IFN-γ) is mainly secreted by natural killer (NK) cells and activated T cells and plays important roles in regulation of host antiviral responses [16]. Type II IFN induces phosphorylation and activation of JAK1 and JAK2 by interacting with the IFN-γ receptor, which is composed of IFNGR1 and IFNGR2 subunits [17]. The activated JAK1 and JAK2 further phosphorylate STAT1 to form homodimers naming gamma activated factor (GAF), which is transported into the nucleus and binds to the gamma interferon activated site (GAS) elements to initiate transcription of IFN-γ-regulated antiviral genes which play a significant role in host innate immune response against pathogen infection [9,11,18].

Paramyxoviruses have evolved multiple strategies to counteract IFN-meditated antiviral effect through different viral proteins. The P gene products of paramyxoviruses have been implicated in blocking host IFN response, such as the V protein degrades STATs and inhibits the phosphorylation of STATs, blocking STATs’ nuclear translocation [19,20,21,22,23]. The PPRV V protein blocks STAT1 and STAT2 nuclear translocation through interacting with STAT1 and STAT2 [24]. Recently, it has been described that the N proteins of the measles virus (MeV), nipah virus (NiV), and hendra virus (HeV) inhibit IFN responses through different mechanisms [25,26]. In this study, N and P proteins were identified as two novel IFN antagonistic proteins of PPRV. Both N and P protein showed inhibitory effects on the ISRE and GAS promoter activation induced by IFN-β and IFN-γ, respectively. The expression of antiviral genes induced by IFN-β or IFN-γ was also considerably suppressed by N and P proteins. Further investigation indicated that the nuclear translocation of STAT1 was blocked by N and P protein suppression that, in turn, impaired the expression of various antiviral genes. 

## 2. Materials and Methods

### 2.1. Cells, Virus, and Antibodies

Human embryonic kidney 293T cells (HEK-293T) and human epithelial carcinoma cells (Hela) were grown in DMEM/high glucose medium (Gibco, Grand Island, NY, USA) supplemented with 10% fetal bovine serum (FBS; Biological Industries, Israel) and 1% penicillin–streptomycin (Gibco) at 37 °C in 5% CO_2_. The PPRV strain Nigeria 75/1 (GenBank: X74443) was used in this study. The HEK-293T cells were grown to ~50% confluency and then infected with 0.1 MOI of PPRV and maintained in the DMEM medium supplemented with 1% FBS for different times. The infected cells were treated with IFN and subjected to subsequent analysis. The commercial antibodies used in this study were as follows: anti-Flag mouse monoclonal antibody (Sigma–Aldrich, St. Louis, MO, USA), anti-Myc mouse monoclonal antibody (Santa Cruz Biotechnology, Santa Cruz, CA, USA), anti-STAT1 antibody (Santa Cruz Biotechnology), and anti-β-Actin antibody (Thermo Fisher Scientific, USA); rabbit antibodies including anti-DDDDK (FLAG equivalent) (Proteintech, Rosemont, IL, USA), anti-STAT1 rabbit polyclonal antibody (Cell Signaling Technology, Danvers, MA, USA), anti-STAT2 rabbit polyclonal antibody (Cell Signaling Technology), anti-p–STAT1 rabbit polyclonal antibody (Cell Signaling Technology), and anti-p–STAT2 rabbit polyclonal antibody (Cell Signaling Technology). Alexa Fluor^®^488-conjugated goat anti-rabbit and Alexa Fluor^®^594-conjugated goat anti-mouse IgG (H+L) secondary antibodies were purchased from Thermo Fisher Scientific. HRP-conjugated goat anti-rabbit IgG (H+L) and goat anti-mouse IgG (L chain) were obtained from Thermo Scientific and Abbkine Scientific (Wuhan, China).

### 2.2. Plasmids Transfection and Luciferase Reporter Assays

The cDNA encoding for viral proteins of PPRV strain Nigeria 75/1 was reverse transcribed from PPRV RNA using M-MLV (Invitrogen, Grand Island, NY, USA) and cloned into p3xFLAG-CMV-7.1 mammalian expression vector (Sigma–Aldrich). The cDNAs of JAK1, JAK2, TYK2, STAT1, STAT2, and IRF9 were obtained from inserts previously cloned in pcDNA3.1/myc-His(-) vector (Thermo Fisher Scientific). P mutants were constructed by site-directed mutagenesis PCR. The region of amino acids 1–375 of PPRV N protein was defined as the N-core domain, and the 376–525 region was defined as N-tail domain. N-core domain and N-tail domains of PPRV N gene were synthesized and cloned into p3xFLAG-CMV-7.1 vector using GenScript (Nanjing, China). Inserts of plasmid constructs were confirmed by DNA sequencing. The pISRE-Luc, pGAS-Luc, and pRL-TK control plasmids were purchased from Promega (Madison, WI, USA). Primers used for plasmids construction and their corresponding sequences are listed in Table 1.

The HEK-293T cells were transfected with reporter plasmids along with indicated plasmids coding for viral proteins using Lipofectamine 2000 Transfection Reagent (Thermo Fisher Scientific) according to the manufacturer’s instructions. At 24 h post-transfection, cells were treated with IFN-β (1000 U/mL) (R&D Systems, Minneapolis, MN, USA) or IFN-γ (100 ng/mL) (PeproTech, Rocky Hill, NJ, USA) for 12 h. Next, the treated cells were lysed using passive cell lysis buffer (Promega) and luciferase activities were evaluated using a Dual-Luciferase^®^ Reporter Assay System (Promega). The pRL-TK plasmids were co-transfected as controls.

### 2.3. Co-Immunoprecipitation Assays and Western Blotting Analysis

Cells were washed with phosphate-buffered saline (PBS) and lysed using NP40 cell lysis buffer (20 mM Tris-HCl, 150 mM NaCl, 1 mM EDTA, 1% Nonidet P-40) supplemented with a protease and phosphatase inhibitor cocktail (Thermo Fisher Scientific) for 30 min at 4 °C. Supernatants of cell lysates were incubated with anti-Flag antibody (Sigma–Aldrich) at 4 °C overnight. Protein G-Sepharose beads (GE HealthCare, Chicago, IL, USA) were added and incubated for 1.5 h. Immunoprecipitates were subjected to Western blotting analysis. For Western blotting analysis, the protein samples were separated via 10% SDS-PAGE and transferred to nitrocellulose membranes (Merck Millipore, Burlington, MA, USA). Membranes were blocked by 5% skimmed milk for 1 h and followed by incubation with appropriate antibodies at 4 °C overnight. The membranes were exposed by chemiluminescence reagents (Thermo Fisher Scientific) as described previously [27]. 

### 2.4. Indirect Immunofluorescence Assays

Cells were seeded into Nunc^TM^ glass bottom dishes and transfected with various plasmids using Lipofectamine^TM^2000 according to the manufacturer’s instruction. At 24 h post-transfection, the cells were stimulated with IFN-β (2000 U/mL) or IFN-γ (200 ng/mL) for 30 min. The cells were fixed and permeabilized as previously described [28]. After blocking with 5% bovine serum, the cells were incubated with appropriate primary antibodies and fluorochrome-conjugated secondary antibodies, respectively. Nuclei were visualized after addition of ProLong^®^ Gold Antifade Reagent containing 4′,6-diamidino-2-phenylindole (DAPI) (Thermo Fisher Scientific), and the cells were then observed using confocal microscopy (Leica Microsystems, Wetzlar, Germany). All images were captured and processed by Leica Application Suite X (Version 1.0. Leica Microsystems). 

### 2.5. RNA Isolation and Quantitative Real-Time PCR (qPCR)

Total RNA was extracted from cells using TRIzol Reagent (Thermo Fisher Scientific) following the manufacturer’s protocol. The cDNA was reverse transcribed using M-MLV (Thermo Fisher Scientific) and random hexamers, and TB Green Premix ExTaq Reagent (Takara, Dalian, China) were used for real-time quantitative PCR using a QuantStudio 5 Real-Time QPCR System (Applied Biosystems, Waltham, MA, USA) as described previously [29]. Transcript levels of the gene for glyceraldehyde-3-phosphate dehydrogenase (GAPDH) were determined to normalize total RNA input. Relative gene expression was evaluated using the 2^-△△CT^ method. All specific primers in this study were listed in Table 2.

### 2.6. Statistical Analysis

All data are presented as mean ± SEM with an error bar that represents at least three independent experiments. Statistical analysis was performed using GraphPad Prism version 5.0 (GraphPad Software, San Diego, CA, USA). Differences in indicators between treatment groups and controls were assessed using the Student’s *t*-test. A two-tailed *p-*value < 0.05 was considered statistically significant and are marked as “*” in the legend. *p* < 0.01 and *p* < 0.001 values are marked as “**” and “***” to indicate corresponding results were highly significant.

## 3. Results

### 3.1. PPRV N and P Proteins Inhibit Both IFN-β- and IFN-γ-Induced IFN Response

**Figure 1:**
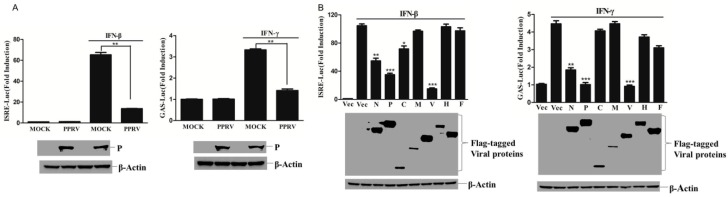

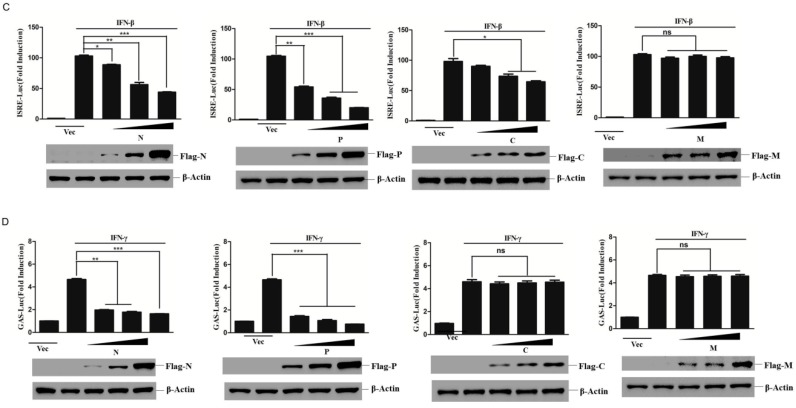
PPRV N and P proteins suppressed IFN-β and IFN-γ induced response. (**A**) HEK-293T cells in 24 well culture plates were transfected with ISRE (100 ng) or GAS (100 ng) reporter plasmids along with pRL-TK (10 ng) plasmid and mock-infected or infected by PPRV (0.1 MOI) at 6 h post-transfection (hpt) for another 48 h. The infected cells were then treated with IFN-β (1000 U/mL) or IFN-γ (100 ng/mL). Luciferase activity was measured at 12 h after IFN-β or IFN-γ treatment. The expression of viral P protein was detected by Western blotting and used as an infection indicator. (**B**) HEK-293T cells in 24 well culture plates were transfected with ISRE (100 ng) or GAS (100 ng) reporter plasmids together with pRL-TK (10 ng) plasmids and 200 ng of various PPRV viral protein expressing plasmids or vector plasmids. At 24 h post-transfection, the cells were treated or mock treated with IFN-β (1000 U/mL) or IFN-γ (100 ng/mL). Luciferase activity was determined at 12 h after IFN-β or IFN-γ treatment. The expression of various PPRV proteins in the transfected cells were detected by Western blot analysis using anti-Flag tag antibody. (**C** and **D**) HEK-293T cells in 24 well culture plates were transfected with gradient dose of plasmids coding for N, P, C or M (100, 200 or 300 ng) along with ISRE (100 ng) or GAS (100 ng) reporter plasmids and pRL-TK (10 ng) plasmids for 24 h. Then the cells were treated with IFN-β (1000 U/mL) or IFN-γ (100 ng/mL) for 12 h and subjected to luciferase activity determination. The expression of the viral proteins was detected by Western blotting.

The PPRV infection resulted in acute immune suppression [30]. To investigate whether PPRV infection inhibits IFN-β or IFN-γ responses, IFN-β- or IFN-γ-induced ISRE and GAS promoter activation status in PPRV-infected cells was evaluated. The IFN-β-induced ISRE promoter activation and IFN-γ-induced GAS promoter activation were both significantly inhibited by PPRV infection (Figure 1A). To identify the viral proteins that were responsible for the PPRV-mediated antagonistic role against IFN-β- and IFN-γ-induced response, the plasmids expressing various viral proteins were co-transfected with ISRE or GAS reporter plasmids, and the cells were then mock-treated or treated with IFN-β and IFN-γ. The results showed that IFN-β incubation considerably activated ISRE promoter activation and IFN-γ treatment highly activated GAS promoter activation. However, PPRV N, P, and V proteins significantly impaired the activation of both ISRE promoter activation and GAS promoter activation (Figure 1B). The expression of these viral proteins was confirmed by Western blot analysis (Figure 1B). The antagonistic role of V protein has been reported previously. Therefore, we further focused our study on the antagonistic effect of N and P proteins. The dose-dependent assays demonstrated that both N and P proteins inhibited the ISRE and GAS promoter activation in a dose-dependent manner. However, C protein only slightly suppressed the ISRE promoter activation. The M protein failed to inhibit ISRE or GAS promoter activation (Figure 1C,D). These results indicate that PPRV N and P proteins play an antagonistic role against both IFN-β- and IFN-γ-induced IFN response.

### 3.2. PPRV N and P Proteins Suppress IFN-β- and IFN-γ-Induced Expression of Antiviral Genes

**Figure 2:**
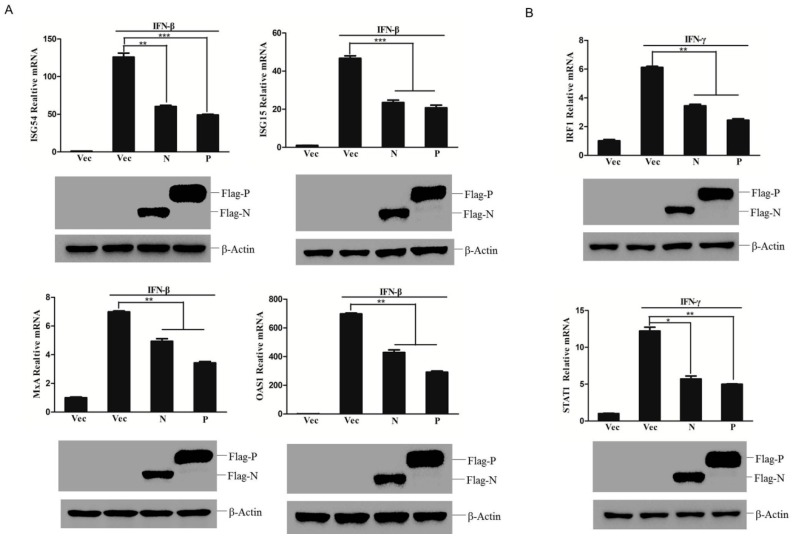
PPRV N and P proteins inhibited the expression of IFN-β or IFN-γ-induced antiviral genes. HEK-293T cells grown in 6 well culture plates were transfected with N protein expressing plasmids (2 μg), P protein expressing plasmids (2 μg) or vector plasmids (2 μg). The cells were treated with IFN-β (1000 U/mL) (**A**) or IFN-γ (100 ng/mL) (**B**) for 12 h at 24 h post-transfection. qPCR analysis of various antiviral genes was performed to determine the relative mRNA expression levels. The expression of the viral proteins was detected by Western blotting.

IFN induces expression of a crucial series of host cellular genes that perform a variety of functions via their antiviral, immune-modulatory, and antitumor activities [9]. Some of these genes are commonly inducible by both IFN-β and IFN-γ, while others are only selectively induced by either IFN-β or IFN-γ. IFN-β initiates upregulation of a set of downstream ISGs, such as *ISG54*, *ISG15*, *OAS1,* and *MxA*, which play key roles in the antiviral response. To determine whether PPRV N and P proteins inhibited expression of ISGs induced by IFN-β, mRNA levels of *ISG54*, *ISG15*, *OAS1,* and *MxA* were measured in the IFN-β-treated cells which had been transfected with empty vector, N or P expressing plasmids. IFN-β treatment induced high expression of these ISGs in the empty vector plasmids transfected cells. However, in the N or P expressing plasmid transfected cells, the expression of these ISGs were remarkably decreased (Figure 2A). Similarly, IFN-γ induced the expression of IFN-γ-regulated genes including *IRF1* and *STAT1*. *IRF1* is thought to coordinate the expression of multiple inflammatory genes and cytokines, and *STAT1* is an important gene to regulate signal transduction [17]. Overexpression of N or P proteins significantly inhibited IFN-γ-induced upregulation of *IRF1* and *STAT1* (Figure 2B). These results suggest that PPRV N and P proteins inhibit the expression of antiviral related genes induced by both IFN-β and IFN-γ.

### 3.3. PPRV N and P Proteins Block STAT1 Nuclear Translocation

**Figure 3:**
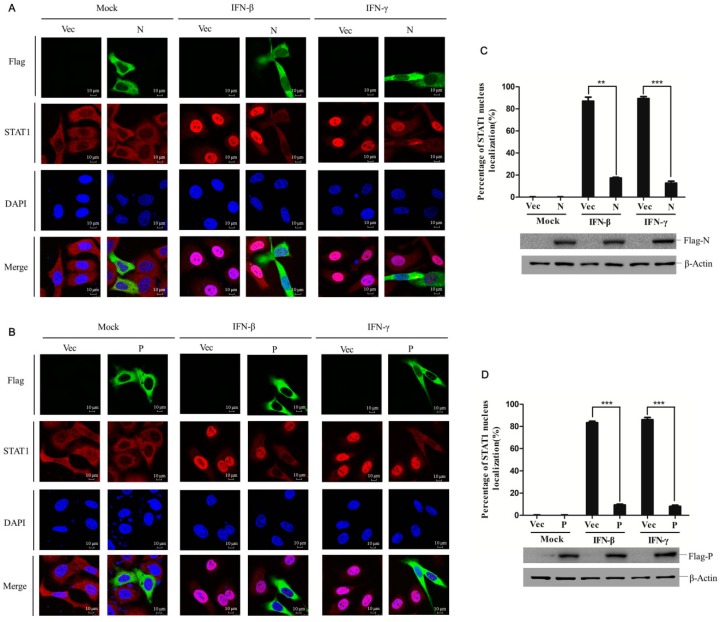
PPRV N and P proteins inhibited STAT1 nuclear translocation. (**A** and **B**) HeLa cells (5 × 10^4^) were grown in the glass bottom dish. When the cells reached about 30% confluency, the cells were transfected with N protein expressing plasmids (2 μg), P protein expressing plasmids (2 μg) or vector plasmids (2 μg), followed by IFN-β (2000 U/mL) or IFN-γ (200 ng/mL) treatment for 30 min. The cells were fixed, permeabilized, and incubated with mouse anti-STAT1 antibody and rabbit anti-Flag antibody and appropriate secondary antibodies. The subcellular localization of STAT1 in N protein overexpressing cells (**A**) or P protein overexpressing cells (**B**) was observed (Red). Confocal images were obtained using laser confocal microscopy under a 60× objective. (**C**) Statistical analysis of STAT1 nuclear translocation in the N and P protein expressing cells. One hundred cells were counted in the randomly selected visual fields three times, and the STAT1 nuclear localization ratio was determined. The expression of N and P was further detected by Western blotting.

STAT1 nuclear transportation is an essential step for downstream gene activation during IFNs signaling cascades, regardless of IFN type. Because PPRV N and P proteins block both IFN-β- and IFN-γ-induced signaling, it would be interesting to determine if N or P proteins block nuclear translocation of STAT1 via subcellular localization of STAT1 within N- and P-overexpressing cells stimulated with IFN-β or IFN-γ. HeLa cells were transfected with vector plasmids, N- or P-expressing plasmids for 24 h, and then mock-treated or treated with IFN-β or IFN-γ for 30 min. In the vector transfected cells, STAT1 mainly distributed into the cytoplasm (Figure 3A,B), and IFN-β or IFN-γ treatment considerably induced STAT1 nuclear transportation. However, in the N- or P-expressing cells, no matter IFN-β or IFN-γ treatment, a large amount of STAT1 remained distributed into the cytoplasm (Figure 3A,B). A statistical evaluation was used to analyze the subcellular localization status of STAT1 in the nucleus and cytoplasm, which confirmed the suppressive role of N and P proteins on STAT1 nuclear transportation (Figure 3C,D). The expression of N and P proteins was also detected by Western blotting (Figure 3C,D). These results indicate that PPRV N and P proteins significantly blocked the nuclear transportation of STAT1 stimulated by IFN-β or IFN-γ.

### 3.4. PPRV N Protein Did Not Block Phosphorylation and Dimerization of STATs

**Figure 4:**
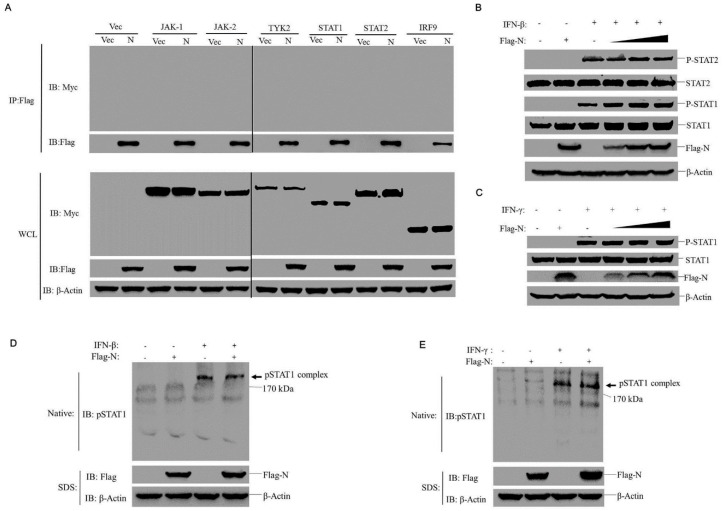
N protein did not interact with molecules of the JAK-STAT pathway or inhibit STAT’s activation. (**A**) HEK-293T cells were transfected with Flag-N expressing plasmids and Myc-tagged JAK1, JAK2, TYK2, STAT1, STAT2, and IRF9 expressing plasmids. The cells were lysed and immunoprecipitated with anti-Flag antibody. Immunoprecipitation (IP) and whole-cell lysates were analyzed by immunoblotting (IB) using anti-Myc, anti-Flag, and anti-β-actin antibodies. (**B** and **C**) HEK-293T cells were transfected with increasing amounts of N protein expressing plasmids (1, 2 or 3 μg). The cells were treated with IFN-β (1000 U/mL) (**B**) or IFN-γ (100 ng/mL) (**C**) at 24 h post-transfection for 30 min. The cell lysis was subjected to Western blotting analysis using anti-Flag, anti-p–STAT1, anti-p–STAT2, anti-STAT1, anti-STAT2, and anti-β-Actin antibodies, respectively. (**D** and **E**) HEK-293T cells were transfected with 2 μg of N protein expressing plasmids for 24 h. The cells were then treated with IFN-β (1000 U/mL) (**D**) or IFN-γ (100 ng/mL) (**E**) for 30 min. Cells were lysed and subjected to Native-PAGE analysis and were detected using anti-pSTAT1 antibody, the expression of N and β-actin was subjected to SDS-PAGE and detected using appropriate antibodies.

Since PPRV N and P proteins inhibited STAT1 nuclear transport, we next investigated JAK-STAT pathway cascades that might be targeted by N protein (the most abundant protein of PRRV). To determine which step was affected by N protein, we examined whether the N protein interacted or degraded one or several important signal molecules of the JAK-STAT pathway. The HEK-293T cells were co-transfected with Flag-tagged N protein expressing plasmids or vector plasmids along with a series of plasmids expressing Myc-tagged JAK1, JAK2, TYK2, STAT1, STAT2, or IRF9. Cell lysates were immunoprecipitated using anti-Flag antibody and then subjected to Western blotting analysis. As shown in Figure 4A, no N protein interaction with any of the adaptor molecules was observed and N protein expression did not inhibit expression levels of these adaptors. Next, because STAT1 phosphorylation triggered by IFN-β or IFN-γ normally leads to STATs dimerization, an essential step for STAT1 nuclear transport, we investigated whether N protein affected the phosphorylation STAT1 and STAT2 or STATs dimerization induced by IFN-β or IFN-γ. The HEK-293T cells were transfected with increasing amounts of N protein-expressing plasmids and then treated with IFN-β or IFN-γ. As shown in Figure 4B, the phosphorylation of STAT1 or STAT2 stimulated by IFN-β was not affected by expression of N protein. The phosphorylation of STAT1 stimulated by IFN-γ was not affected by expression of N protein as well (Figure 4C). Meanwhile, evaluation of STAT’s dimerization showed that overexpression of N protein also had no effect on STAT’s dimerization induced by IFN-β or IFN-γ (Figure 4D and E). It suggests that N protein has no influence on the phosphorylation and dimerization of STATs.

### 3.5. Core and Tail Domains of N Protein Play Different Suppressive Roles on IFN-β- and IFN-γ-Triggered Cellular IFN Responses

**Figure 5:**
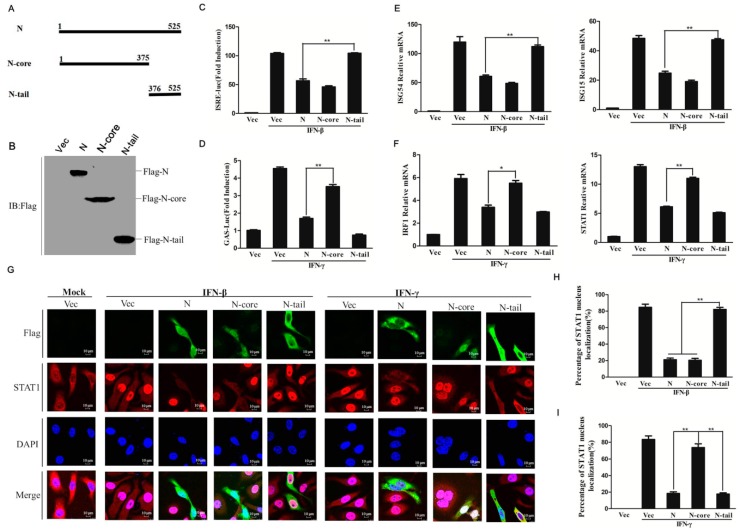
N-core domain and N-tail domain of PPRV N protein show different antagonistic roles in IFN response inhibition. (**A**) Schematics of N, N-core, and N-tail. (**B**) The expression of N-core and N-tail domain in HEK-293T cells were analyzed by immunoblotting. (**C** and **D**) HEK-293T cells were co-transfected with ISRE (**C**) or GAS (**D**) reporter plasmids together with pRL-TK plasmids, and empty vector, N, N-core or N-tail expressing plasmids. The IFN treatment and luciferase activity detection were performed at 24 h post-transfection as described in Figure 1B. (**E** and **F**) HEK-293T cells were transfected with empty vector, N, N-core or N-tail expressing plasmids for 24 h. The cells were then treated with IFN-β (E) or IFN-γ (F) for 12 h. The relative mRNA expression levels of *ISG54*, *ISG15*, *IRF1*, and *STAT1* were determined respectively. (**G**) HeLa cells (5 × 10^4^) were grown in the glass bottom dish. The monolayer cells were transfected with N (2 μg), N-core (2 μg), N-tail domain expressing plasmids (2 μg) or vector plasmids (2 μg), followed by IFN-β (2000 U/mL) or IFN-γ (200 ng/mL) treatment for 30 min. The cells were fixed and permeabilized and incubated with mouse anti-STAT1 antibody and rabbit anti-Flag antibody and appropriate secondary antibodies. The subcellular localization of STAT1 was detected (Red). Confocal images were obtained using laser confocal microscopy under a 60× objective. (**H** and **I**) Statistical analysis of STAT1 nuclear translocation in the N-core and N-tail proteins expressing cells. One-hundred cells were counted in the randomly selected visual fields for three times, and the STAT1 nuclear localization ratio was determined.

Paramyxovirus N proteins each possess an N-terminal highly conserved helical-core domain and a C-terminal disordered tail domain [31,32,33]. To determine which N protein domain was responsible for inhibition of IFN-β- and IFN-γ-induced responses observed in this study, two truncated mutants expressing the core domain and tail domain of N protein were constructed, respectively (Figure 5A). Expression of each truncation was confirmed by Western blot analysis (Figure 5B). The effects of core domain (N-core) and tail domain (N-tail) on IFN responses were separately evaluated. As shown in Figure 5C, the N-core domain inhibited IFN-β-induced ISRE promoter activation, while the N-tail domain did not. By contrast, the N-tail domain exerted a crucial suppressive effect upon IFN-γ-induced GAS promoter activation, while the N-core domain exhibited no inhibitory effect (Figure 5D). The suppressive roles of both mutants were further investigated by evaluating their effects on expression levels of various host antiviral genes normally induced by IFN-β or IFN-γ. As observed in the aforementioned reporter assay, IFN-β-induced expression of *ISG54* and *ISG15* was significantly inhibited by the N-core domain, but not by the N-tail domain (Figure 5E), while IFN-γ-induced expression of *IRF1* and *STAT1* was inhibited by the N-tail domain, but not by the N-core domain (Figure 5F). The effect of N-core and N-tail on nuclear transport of STAT1 was also investigated by IFA assay (Figure 5G), which showed that N-core suppressed the nuclear import of STAT1 induced by IFN-β but not IFN-γ. However, N-tail only suppressed IFN-γ-induced nuclear import of STAT1. A statistical evaluation was used to analyze the subcellular localization status of STAT1 in the nucleus and cytoplasm which confirmed the different effect of N-core and N-tail (Figure 5H and I). These results suggest that PPRV N protein core and tail domains play different antagonistic roles and each operate in an IFN type-specific manner.

### 3.6. PPRV P Protein Interacts with STAT1 and Blocks STAT1 phosphorylation

**Figure 6:**
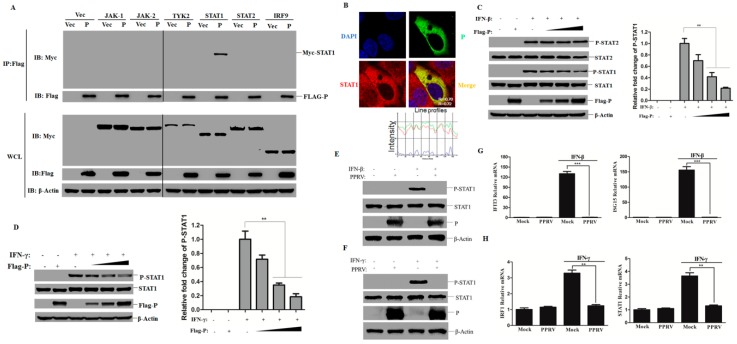
PPRV P protein binds to STAT1 and inhibits STAT1 phosphorylation. (**A**) HEK-293T cells were transfected with Flag-P expressing plasmids and Myc-tagged JAK1, JAK2, TYK2, STAT1, STAT2, and IRF9 expressing plasmids. The cells were lysed and subjected to immunoprecipitation and immunoblotting analysis similar as described in Figure 4A. (**B**) HeLa cells were transfected with P protein expressing plasmids for 24 h, the colocalization of P (Green) and STAT1 (Red) was determined by IFA, the nucleus was stained with DAPI (Blue). The overlapping coefficient (*r*) and Pearson’s correlation (*R*^2^) values were further determined in the merged image. The intensity profile of the linear region of interest (ROI) across a HeLa cell co-stained with P and STAT1 is presented below the merged image. (**C** and **D**) HEK-293T cells were transfected with increasing amounts of P protein expressing plasmids (1, 2 or 3 μg). The cells were treated with IFN-β (1000 U/mL) (**C**) or IFN-γ (100 ng/mL) (**D**) at 24 h post-transfection for 30 min. The relative fold change of p–STAT1 was determined by densitometric analysis after being normalized to β-actin. The cell lysis was subjected to Western blot analysis using anti-Flag, anti-p–STAT1, anti-p–STAT2, anti-STAT1, anti-STAT2, and anti-β-Actin antibodies, respectively. (**E** and **F**) HEK-293T cells were infected with PPRV for 48 h and then treated with IFN-β (1000 U/mL) (**E**) or IFN-γ (100 ng/mL) (**F**) for 30 min. The cells were then lysed and subjected to immunoblotting analysis using the indicated antibodies against pSTAT1, STAT1, P, and β-Actin. (**G** and **H**) Goat fibroblasts were infected with PPRV for 36 h and then the cells were treated with IFN-β (1000 U/mL) (**G**) or IFN-γ (100 ng/mL) (**H**) for 12 h. The qPCR analysis of various antiviral genes was performed to determine the relative mRNA expression levels.

Besides N protein, we also investigated whether the PPRV P protein targeted adaptor molecules of the JAK-STAT pathway. As demonstrated in Figure 6A, only STAT1 co-precipitated with P protein, while P protein showed no inhibitory effect on the expression of these adaptor molecules. Moreover, a clear cytoplastic co-localization pattern between P protein and endogenous STAT1 was observed as well, with most endogenous STAT1 localizing to the cytoplasm (Figure 6B). The overlapping coefficient (*r*) and Pearson’s correlation (*R*^2^) values were further determined, which showed the significant colocalization of STAT1 and P protein (Figure 6B). The effect of P protein on STAT’s phosphorylation was subsequently evaluated. The phosphorylation of STAT1 induced by either IFN-β or IFN-γ was significantly blocked by the presence of P protein in a dose-dependent manner (Figure 6C,D). The relative fold change of p–STAT1 was also determined by densitometric analysis after normalized to β-actin which confirmed this inhibitive effect by P protein (Figure 6C,D). However, phosphorylation of STAT2 induced by IFN-β was not affected by P protein (Figure 6C). The STAT1 phosphorylation induced by IFN-β or IFN-γ was also detected in the PPRV-infected cells. Similarly, the phosphorylation of STAT1 was dramatically impaired by PPRV infection, no matter treated with IFN-β or IFN-γ (Figure 6E,F). The expression of ISGs in the goat fibroblasts that was infected by PPRV and treated with IFN-β or IFN-γ was further detected. The results showed that PPRV infection dramatically decreased the downstream antiviral genes regulated by STAT1 (Figure 6G and H). Collectively, these results indicate that P protein interacts with STAT1 and inhibits IFN-β- and IFN-γ-induced STAT1 phosphorylation and resulted in decreased expression of ISGs.

### 3.7. Tyrosine 110 of P Protein is Critical for Blocking IFN-β- and IFN-γ-Induced STAT1 Activation

**Figure 7:**
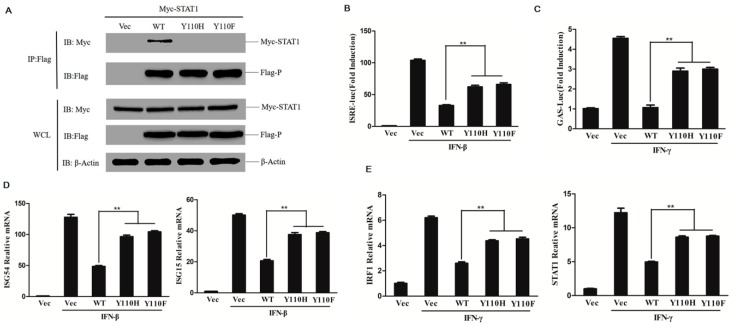
Tyrosine 110 of the P protein is a key site for P–STAT1 interaction and inhibition of IFN response. (**A**) HEK-293T cells were co-transfected with Myc-tagged STAT1 expression plasmids and empty vector, P protein, Y110H mutant or Y110F mutant. Immunoprecipitation (IP) and whole-cell lysates were analyzed by immunoblotting (IB) using anti-Myc, anti-Flag, and anti-β-actin antibodies. (**B** and **C**) HEK-293T cells were transfected ISRE (**B**) or GAS (**C**) reporter plasmids along with pRL-TK plasmids, and P protein expressing plasmids, Y110H or Y110F. The cells were treated with IFN-β (1000 U/mL) or IFN-γ (100 ng/mL) for 12 h at 24 h post-transfection, and the luciferase activity was determined. (**D** and **E**) HEK-293T cells were transfected with empty vector or P protein expressing plasmids, Y110H or Y110F, and the cells were treated with IFN-β (1000 U/mL) (**D**) or IFN-γ (100 ng/mL) (**E**) for 12 h at 24 h post-transfection. The relative mRNA expression levels of *ISG54*, *ISG15*, *IRF1,* and *STAT1* were measured by qPCR analysis.

P and V proteins of paramyxoviruses share similar N-terminal domains. However, it has been found that these proteins may act via different antagonistic mechanisms to counter host antiviral responses, such as the V protein of rinderpest virus (RPV) could effectively block the IFN-induced phosphorylation of STAT1; however, P protein was relatively inefficient [22,34]. Measles virus (MV) P but not V protein could inhibit STAT1 phosphorylation [35,36]. We determined that PPRV P protein significantly blocked both IFN-β- and IFN-γ-induced phosphorylation of STAT1. A previous study had indicated that tyrosine 110 (Y110) of the PPRV V protein, the key amino acid involved in the interaction between V and STAT1, was involved in inhibiting IFN signaling [24]. We also investigated whether the Y110 of PPRV P protein was involved in the P protein-mediated antagonistic effect involving STAT1 observed here. To avoid a P protein conformational change-mediated explanation for loss of function resulting from amino acid substitution, Y110 was substituted with either a basic amino acid residue (histidine, H) or an aromatic amino acid residue (phenylalanine, F). To investigate whether the mutation of Y110 affects the interaction of P protein with STAT1, Myc-STAT1 expressing plasmids were co-transfected with wild-type P protein (WT), the mutants of P protein Y110H or Y110F expressing plasmids or empty vector in HEK-293T cells. The co-immunoprecipitation assay was then performed. As shown in Figure 7A, introduction of Y110H or Y110F into the P protein abrogated its interaction with STAT1, suggesting that Y110 is a key residue involved in the P protein interaction with STAT1. The V protein shares the N-terminus to the P protein. It suggested both the N terminus of V and P proteins showed significant antagonistic roles, and the involved antagonistic mechanism by the N terminus might be similar.

The effect of P mutants Y110H and Y110F on IFN-β- or IFN-γ-induced IFN response was further investigated. The plasmids expressing mutants Y110H, Y110F or WT P proteins were co-transfected with reporter plasmids and subjected to IFN-β or IFN-γ treatment. The luciferase activity was then measured and compared. The ISRE promoter activation level induced by IFN-β and the GAS promoter activation level induced by IFN-γ were clearly higher in the Y110H or Y110F mutant expressing cells than that in the WT P protein expressing cells (Figure 7B and C). However, the mutation of Y110 did not fully recover the total (100%) activity. The ISRE promoter activation level induced by IFN-β in the Y110H or Y110F mutant expressing cells did not reach the control levels. Maybe other sites were also involved in this suppressive effect. The expression of antiviral-related genes stimulated by IFN-β or IFN-γ were further evaluated and analyzed. The IFN-β-induced expression of *ISG54* and *ISG15* and the IFN-γ-induced expression of *IRF1* and *STAT1* were remarkably increased in the Y110H or Y110F mutant expressing cells than in the WT P protein expressing cells (Figure 7D and E). These results indicate that P protein Y110 plays an important role in inhibiting the IFN-β- and IFN-γ-induced IFN response.

## 4. Discussion

The IFNs elicit host antiviral actions by activating the JAK-STAT pathway and inducing transcription of hundreds of antiviral genes [37]. To evade IFN-mediated antiviral effects, many viruses have evolved various strategies to impair activation of the JAK-STAT pathway through its viral protein. The V protein of PPRV inhibits the IFN response through interaction with STAT1/2 and blocking STAT1/2 nuclear translocation [24]. Recently, it has been demonstrated that the V and C proteins of PPRV inhibited the induction of IFN-β [38], indicating that PPRV may suppress the host antiviral response through different mechanisms mediated by different viral proteins. In this study, we identified that PPRV N and P proteins performed significant antagonistic effect against host IFN response, and they have been shown to counteract the cellular IFN response by targeting the JAK-STAT signaling pathway.

The N proteins of enveloped RNA viruses play an important role in the formation and protection of viral ribonucleoprotein (RNP) complexes. In addition to their functions as structural components, N proteins also counteract host antiviral responses and promote viral replication. In coronaviruses and arenaviruses, N protein has been identified as an antagonistic factor to inhibit IFN production. For instance, porcine epidemic diarrhea virus N protein inhibits IFN production by interacting with TANK binding kinase 1 (TBK1) and disrupting the association of interferon regulation factor 3 (IRF3) with TBK1 [39]. Mouse hepatitis virus (MHV) and severe acute respiratory syndrome coronavirus (SARS-CoV) N protein interacts with the protein activator of protein kinase R (PACT) and disrupts the interaction between PACT and retinoic acid-induced gene I (RIG-I)/melanoma differentiation gene 5 (MDA5), which suppresses the production of IFN [40]. The SARS-CoV N protein also directly interacts with tripartite motif protein 25 (TRIM25) and sequesters the interwork between TRIM25 and RIG-I, and further disturbs the RIG-I ubiquitination mediated by TRIM25 [41]. Arenavirus N protein also interacts with PACT and inhibits PACT-enhanced RIG-I-induced IFN production similar to MHV and SARS-CoV. However, the involved mechanism is different from the antagonistic manner of MHV and SARS-CoV N proteins. Arenavirus N protein performs the suppressive role through its RNase activity [42]. In paramyxoviruses, MV N protein blocks the STAT1/2 nuclear translocation induced by IFNs. The MV N protein blocks the nuclear transportation of STAT without preventing STAT and JAK activation. However, MV N protein acts as an IFN-α/β and γ-antagonist as strong as P protein [25]. NiV and HeV N proteins interfere with the formation of IFN-induced STAT complexes to inhibit the host antiviral response. The NiV and HeV N proteins inhibit the IFN response by interfering with the STAT complex formation in their core domains. The NiV N protein inhibits the nuclear transportation of both STAT1 and STAT2, which is not associated with the nuclear transport system for STATs [26]. In this study, we found that PPRV N protein also impaired the host IFN response by blocking JAK-STAT pathway signaling. No interaction was observed between PPRV N protein and components of the JAK-STAT pathway, and N protein exhibited no inhibitive effect on IFN-β- or IFN-γ-induced phosphorylation of STATs. The PPRV N protein considerably reduced STAT1 nuclear accumulation which was similar than the effect caused by MV and NiV N proteins [25,26]. However, PPRV N protein did not affect STAT’s complex formation. Further studies should be performed to investigate the different mechanisms used by N proteins of various paramyxoviruses.

The N-terminal domain of N protein has previously been identified as the core domain required for RNA binding and nucleocapsid helical core formation [31,32,43]. The C-terminal tail in N protein possesses the features of intrinsically disordered proteins [33]. It is identified that the 1–375 amino acid region of MV N protein is the minimal region for nucleocapsid assembly [31]. In the present study, the region of amino acids 1–375 of the PPRV N protein was defined as the N-core domain, and the 376–525 region was defined as the N-tail domain. We determined that the N-core suppressed the IFN-β-meditated signaling, but the N-tail inhibited the IFN-γ-induced signaling, showing that the different regions of the N protein targeted different IFN pathways. The N-core and N-tail might target different adaptor molecules and result in this distinction. This is different from the N-cores of NiV and HeV, which inhibit both the IFN-β- and IFN-γ-induced IFN response [26]. This might be the reason why N proteins of different paramyxoviruses reveal different functions in suppression of host IFN response. Whether PPRV N protein inhibited the nuclear transport system for STATs should be further investigated. The mechanisms for the different role of N-core and N-tail should also be explored.

The P proteins of paramyxoviruses have two main functions in the viral life cycle: they 1) function as a cofactor of viral RNA-dependent RNA polymerase and 2) serve as a guardian for the correct assembly of nucleocapsids. Meanwhile, the products of P gene in paramyxoviruses have also been shown to inhibit IFN response at various levels [20,22,23,35,44]. In the present study, we determined that PPRV P protein interacted with STAT1 and suppressed STAT1 phosphorylation and nuclear transportation. The P protein of MV was able to interact with STAT1 and prevented the phosphorylation of STAT1 to inhibit the IFN-β-induced response [35,45]. The PPRV P protein may suppress host IFN response through a similar manner used by the MV P protein. However, the MV V protein does not interfere with STAT1 phosphorylation [35,36]. As for the PPRV V protein, it showed an inhibitory effect on STAT1 phosphorylation, which was different from the MV V protein [46]. This also indicated the multiple antagonistic strategies for different paramyxoviruses.

In IFN-β-induced signaling, JAK1 is responsible for STAT1 phosphorylation, while both JAK1 and JAK2 are critical for IFN-γ-induced STAT1 phosphorylation [47]. The PPRV V protein interacts with JAK1 [46], but we did not observe an interaction between P protein and JAK1. Both PPRV P and V proteins interacted with STAT1. The tyrosine 110 (Y110) in the PPRV V protein is critical for its interaction with STAT1. We found mutation of Y110 in the P protein completely abrogated its interaction with STAT1 and resulted in a considerably decreased suppressive effect of P protein on IFN response. The Y110 site of PPRV V and P proteins might share a similar antagonistic mechanism by targeting STAT1. The Y110 in the MV P protein is also required to block STAT1 phosphorylation [35]. The Y110 in the V and P proteins is identified as specific amino acid residue for the STAT1 interaction in some Morbilliviruses [35,48,49]. These results indicate that the Y110 plays a significant role in the V protein and P protein of paramyxoviruses. We speculate that the binding site of STAT1 for PPRV P protein may be essential for phosphorylation of STAT1. The Src homology 2 (SH2) domain of STAT1 is critical for the phosphorylation of STAT1, the P protein of MV binds with the SH2 domain of STAT1, which sequesters the JAK1-meditated STAT1 phosphorylation or hinders the interaction between STAT1 and its receptor [45]. Further investigation will focus on more extensive elucidation of STAT1 residues involved in binding to the PPRV P protein.

## 5. Conclusions

In conclusion, we demonstrated that the N and P proteins functioned as two novel IFN antagonistic factors of PPRV. Multiple viral proteins are involved in suppression of host antiviral response during PPRV infection. Mechanistic studies demonstrate that N and P suppress JAK-STAT pathway signaling by blocking STAT1 nuclear aggregation. Our results definitely broaden our knowledge of PPRV-mediated immune evasion and provide a reference for vaccine development against PPRV.

## Figures and Tables

**Table 1:** The primers used for plasmids construction.

Gene	Primers (5′ → 3′)
JAK1	Forward: ATTT*GCGGCCGC*ATGGCTTTCTGTGCTAAAATG
	Reverse: CGC*GATATC*TTTTAAAAGTGCTTCAAATCCTTC
JAK2	Forward: ATTT*GCGGCCGC*ATGGGGATGGCTTGCCTTACG
	Reverse: GCG*GATATC*TCCAGCCATGTTATCCCTTACTTG
STAT1	Forward: ATTT*GCGGCCGC*ATGTCTCAGTGGTACGAACTT
	Reverse: GCG*GATATC*TACTGTGTTCATCATACTGTC
STAT2	Forward: ATTT*GCGGCCGC*ATGGCGCAGTGGGAAATGCTGC
	Reverse: GCG*GATATC*GAAGTCAGAAGGCATCAAGGGTCC
TYK2	Forward: ATTT*GCGGCCGC*ATGCCTCTGCGCCACTGGGG
	Reverse: GCG*GATATC*GCACACGCTGAACACTGAAGGGGC
IRF9	Forward: ATTT*GCGGCCGC*ATGGCATCAGGCAGGGCACGCT
	Reverse: GCG*GATATC*CACCAGGGACAGAATGGCTGCCTG
N	Forward: TT*GCGGCCGC*GATGGCGACTCTCCTTAAAAGCT
	Reverse: TT*GTCGAC*TCAGCCGAGGAGATCCTTGTCGTT
P	Forward: TT*GCGGCCGC*GATGGCAGAAGAACAAGCATACCAT
	Reverse: GC*GATATC*TTACGGCTGCTTGGCAAGAATGGCTGTTA
C	Forward: CT*GAATTC*AATGTCAACAAGGGACTGGAA
	Reverse: GCC*TCTAGA*CTAATTTTTCGACATCTGTTGACCT
M	Forward: GC*AAGCTT*ATGACCGAGATCTACGA
	Reverse: T*GGATCC*TTACAGGATCTTGAACAGGCCTTGAT
H	Forward: TT*GCGGCCGC*GATGTCCGCACAAAGGGAAAGGAT
	Reverse: GCT*GGTACC*TCAGACTGGATTACATGTTACCTCTATACC
F	Forward: TT*GCGGCCGC*GATGACACGGGTCGCAACCTTAGTATTT
	Reverse: T*GGATCC*CTACAGTGATCTCACGTACGACT
P-Y110H	Forward: AACTCTCAAGTACAGCGT*CAC*TATGTTTATAGCCACGGG
	Reverse: CCCGTGGCTATAAACATA*GTG*ACGCTGTACTTGAGAGTT
P-Y110F	Forward: AACTCTCAAGTACAGCGT*TTC*TATGTTTATAGCCACGGG
	Reverse: CCCGTGGCTATAAACATA*GAA*ACGCTGTACTTGAGAGTT
N-core	Forward: TT*GCGGCCGC*GATGGCGACTCTCCTTAAAAGCT
	Reverse: GCG*GATATC*CTACTTTCCTGCAGATCTTCTG
N-tail	Forward: ATTT*GCGGCCGC*GTCAGCTCTGTAATCGCGGC
	Reverse: TT*GTCGAC*TCAGCCGAGGAGATCCTTGTCGTT

**Table 2:** The qPCR primers used in present study.

Gene	Primers (5′→3′)
*GAPDH*	Forward: CGGGAAGCTTGTGATCAATGG
	Reverse: GGCAGTGATGGCATGGACTG
*ISG54*	Forward: GGTCTCTTCAGCATTTATTGGTG
	Reverse: TGCCGTAGGCTGCTCTCCA
*ISG15*	Forward: TGGACAAATGCGACGAACC
	Reverse: CCCGCTCACTTGCTGCTT
*MXA*	Forward: TCTTCATGCTCCAGACGTAC
	Reverse: CCAGCTGTAGGTGTCCTTG
*OAS1*	Forward: TGTCCAAGGTGGTAAAGGGTG
	Reverse: CCGGCGATTTAACTGATCCTG
*IRF1*	Forward: GAGGAGGTGAAAGACCAGAGCA
	Reverse: TAGCATCTCGGCTGGACTTCGA
*STAT1*	Forward: ATGGCAGTCTGGCGGCTGAATT
	Reverse: CCAAACCAGGCTGGCACAATTG
